# Abdominal Adipose Tissue Associates With Adiponectin and TNFα in Middle-Aged Healthy Men

**DOI:** 10.3389/fendo.2022.874977

**Published:** 2022-07-07

**Authors:** Hani Zaidi, Tonje Aksnes, Sissel Åkra, Heidi B. Eggesbø, Rune Byrkjeland, Ingebjørg Seljeflot, Trine B. Opstad

**Affiliations:** ^1^ Center for Clinical Heart Research, Department of Cardiology, Oslo University Hospital, Ullevål, Norway; ^2^ Faculty of Medicine, University of Oslo, Oslo, Norway; ^3^ Section for Interventional Cardiology, Department of Cardiology, Heart-, Lung-, and Vascular-Disease Clinic, Oslo University Hospital, Oslo, Norway; ^4^ Division of Radiology and Nuclear Medicine, Oslo University Hospital, Oslo, Norway

**Keywords:** adiponectin, TNFα, adipose tissue, gene expression, adipose tissue compartments

## Abstract

**Introduction:**

Adipokines are highly active biopeptides involved in glucose metabolism, insulin regulation and the development and progression of obesity and its associated diseases. It includes, among others, adiponectin, visfatin and tumor necrosis factor alpha (TNFα). The sources of adipokines and their associations with glucometabolic variables are not completely understood.

**Aim:**

In this cross-sectional study, we aimed to investigate whether gene expression levels in subcutaneous adipose tissue (SAT) of selected adipokines and their corresponding circulating levels associate with the amount of AT in superficial (sSAT), deep (dSAT) and visceral AT (VAT), assessed by computed tomography (CT). Any association with glucometabolic variables were also explored.

**Methods:**

In 103 healthy Caucasian men, aged 39.5 years, fasting venous blood and SAT samples from the gluteal region were collected. Ninety-four of the participants underwent CT assessment of the abdominal AT, which was divided into VAT, sSAT and dSAT. Circulating levels of adipokines were measured by ELISA and AT gene-expression by PCR. Insulin sensitivity was determined by glucose clamp, assessing glucose disposal rate (GDR).

**Results:**

Circulating adiponectin and TNFα gene expression correlated inversely and positively to the amount of AT in all three compartments (r=-0.266 to -0.276, p<0.05 for all) and (r=0.323 - 0.368, p<0.05 for all), respectively, with strongest correlations to the amount in sSAT and dSAT. When dividing AT compartments into quartiles, a tendency was observed towards lower circulating adiponectin and higher TNFα gene expression levels, respectively, with increasing amount of sSAT and dSAT. Circulating adiponectin correlated inversely to insulin, C-peptide and waist circumference (r=-456 to -0.373, p<0.001) and positively to GDR (r=0.356, p<0.001). AT-expressed visfatin correlated inversely to insulin and C-peptide (r=-0.370 and r=-0.404, p<0.001).

**Conclusion:**

Increased amount of AT is associated with lower levels of adiponectin and increased levels of TNFα AT expression.

## Introduction

Adipokines are highly active biopeptides produced by adipose tissue (AT), involved in regulating glucose metabolism, insulin function and the development and progression of obesity and its associated diseases (1). Thus, AT also exerts its function in an endocrine manner, which is widely accepted (2). Adipokines have been classified as hormones, cytokines and growth factors and includes, among others, adiponectin, visfatin and tumor necrosis factor alpha (TNFα) (3).

AT may be divided into visceral adipose tissue (VAT) and abdominal subcutaneous AT (SAT), further subdivided into superficial (sSAT) and deep (dSAT). Both SAT and especially VAT have been associated with the metabolic syndrome (MetS) and its risk factors (4). dSAT and VAT are reported to have similar and a more pro-inflammatory profile than sSAT, both associated with insulin resistance and atherosclerosis (5, 6). The mechanism behind this differentiation remains unclear, but it has been speculated whether VAT is a more bioactive compartment than SAT irrespective of BMI (7).

Adiponectin is produced almost exclusively by adipocytes (8). It is known to have insulin sensitizing, anti-atherogenic and anti-inflammatory properties, and hypoadiponectemia is associated with obesity, insulin-resistance and type 2 diabetes mellitus (T2DM) (9, 10). Adiponectin is believed to exert its actions by inhibiting transformation of macrophages into foam cells and through inhibition of TNFα (11). Adiponectin seems furthermore to play a key role in determining metabolic health, independent of body mass index (BMI), as shown in healthy individuals (12).

Visfatin, predominantly expressed in VAT, is elevated in obesity and T2DM (13, 14). Visfatin is particularly interesting to examine in order to understand the biological difference between VAT and SAT, as previous studies have reported associations to the amount of VAT, but not to SAT (15).

TNFα was initially assumed to be produced by adipocytes in adipose tissue, due to its correlation to BMI (16). However, the stromovascular fraction of AT, including endothelial cells, macrophages and leukocytes, seems to have higher TNFα production than adipocytes (17). Fain et al. also demonstrated that it was primarily the non-fat cells in AT that were responsible for TNFα production (18). TNFα is thought to increase insulin resistance and cause dyslipidemia through suppression of adiponectin production (19). Furthermore, TNFα modulates insulin receptors through inhibition of tyrosine kinase activity, decreasing their insulin sensitivity (20).

The relationship between AT distribution and adipokines has not been adequately investigated and we therefore aimed to examine whether gene expression levels in SAT of selected adipokines and their corresponding circulating levels, associates with the amount of abdominal AT in sSAT, dSAT and VAT in a cohort of middle-aged, healthy men. We hoped to gain a better understanding of the source of circulating adipokines. Any associations with glucometabolic variables that have previously been linked to adipokines were further explored.

## Materials and Methods

### Material

The present investigation is based on a 20-year follow-up of an initial study population of 103 healthy Caucasian men, recruited at the enlistment at the age of 20 in Oslo, Norway. Eleven participants used one or more of the following drugs: thyroid replacement drug (n=2), antidepressants (n=3), oral antidiabetic drug (n=1), blood pressure lowering medication (n=5) and cholesterol lowering medication (n=3). The study has been described in detail previously (21). It was conducted in accordance with the Declaration of Helsinki and The Regional Committee of Medical and Health Research Ethics approved the study and informed, written consent was obtained from all participants.

In brief, all participants were previously healthy, and the mean age was 40 years at the time of the present study. In this sub-study, we further analyze the biobank attained from the aforementioned population.

### Methods

#### Computed Tomography

Ninety-two of these participants underwent computed tomography (CT) assessment of the abdominal AT. They were examined in a supine position, arms extended above the head with one single axial scan performed without intravenous contrast medium, through the mid-abdomen, at the L3-L4 level. The circumferences were tracked for sSAT and dSAT compartments, divided by a membranous layer (Scarpa’s fascia) and the muscle compartment including the spine. The VAT compartment was measured by tracking circumferences between the inner abdominal wall and dSAT, which also includes the muscle compartment and the spine, and then highlighting the pixels containing fat. Although measured as area, we have used the term adipose tissue amount in our description. The details of the CT analyses have previously been described (22).

#### Laboratory Methods

Fasting venous blood samples were drawn between 8 and 11 AM at the time of follow-up. Fasting glucose, HbA1c, insulin, C-peptide, cholesterols and triglycerides were determined by conventional routine methods. Insulin sensitivity was assessed with a 120-minute hyperinsulinemic euglycemic glucose clamp, which is a gold standard for measurement of insulin sensitivity (23, 24). A low glucose disposal rate (GDR), has been associated with increased risk of diabetes vascular complications such as retinopathy, cardiovascular disease, nephropathy or a composite of any complication (25). A higher GDR implies better insulin sensitivity. Subcutaneous AT was sampled from the gluteal region, and immediately stored at -80°C until RNA extraction.

Serum was prepared by centrifugation within 1 hour for 10 min at 2500 x g for determination of circulating adiponectin, visfatin and TNFα, measured by the following ELISA methods: Human total Adiponectin/Acrp30 and Human TNFα (both R&D Systems Europe, Abingdon, Oxon, UK) and Human Visfatin (MyBioSource California, USA). The inter-assay coefficients on variation in our laboratory were 4.9%, 4.6% and 9.7%, respectively.

Total RNA from SAT was isolated by use of RNeasy Lipid Tissue Mini Kit and the QIAcube according to the manufacturer protocol. RNA quality and quantity (ng/µL) were determined by the NanoDrop™ 1000 Spectrophotometer (Nanodrop Technologies, DE, USA). Extracted RNA was stored at -80°C until analysis. Copy DNA (cDNA) was synthesized from equal amount of RNA with qScript™ cDNA superMix (Quanta Biosciences Inc., Gaitehersburg, USA). Real-time PCR was performed with TaqMan Low Density Custom Arrays on the ViiA™7 instrument, using TaqMan^®^ Universal PCR Maser Mix (P/N 4324018) and TaqMan^®^assays for adiponectin (Hs00605917_m1), visfatin (Hs00237184_m1) and TNFα(H01113624_g1) (Applied Biosystems, by Life Technologies, Foster City, CA, USA). β-2-microglobulin (Hs99999907_m1) (Applied Biosystems) was used as the endogenous control, and mRNA levels were determined by relative quantification (RQ) using the ΔΔCT method (26).

In **Supplemetary Table 1**, we present an overview of methods for measuring adipokines in different compartments

#### Statistics

Non-parametric tests were mainly used as the distribution of the data was primarily skewed. The demographic data are given as median with 25th and 75th percentiles, unless otherwise stated. Spearmann’s rho was used for correlation analysis and adjusted for by Bonferroni correction. Kruskal-Wallis test was used to observe differences in distribution of markers through quartiles of AT compartments and quartiles of GDR. *P*-values less than 0.05 was considered statistically significant. SPSS version 26 (SPSS Inc., IL, USA) was used for all analyses.

## Results

Characteristics of the study population, the amount of abdominal AT and levels of the measured circulating variables are shown in **Table 1**. The study participants were all male, with an average BMI of 26 kg/m^2^ and an average age of 39.5 years when the blood samples and biopsies were obtained. Sixty-one patients had a BMI above 25 kg/m^2^. Of the 103 participants included in the study, CT scan was available for 92. AT biopsies for adiponectin, visfatin and TNFα were available for 80, 77 and 64 participants, respectively. The excluded samples were mainly due reluctance of some participants to have the procedure performed and due to inadequate tissue sample. Serum sample was available for 102 participants due to 2 lost to follow-up. The glucometabolic variables were in the normal range and 20% had a family history of diabetes. Median GDR was 6.27 mg/kg/min.

**Table 1:** Baseline characteristics, circulating levels and gene expression of the adipokines and the amount of AT in the different abdominal compartments of the study population.

GDR (mg/kg/min)	6.27 (3.79, 8.99)
Fasting glucose (mmol/L)	5.0 (4.8, 5.4)
HbA1c (%)	5.3 (5.1, 5.5)
Insulin (pmol/L)	44 (28, 64)
C-peptide (pmol/L)	641 (511, 851)
BMI (kg/m^2^)	26.0 (23.6, 28.0)
Waist circumference (cm)	93.8 (87.7, 102.6)
Total cholesterol (mmol/L)	5.1 (4.5, 5.7)
HDL cholesterol (mmol/L)	1.31 (1.08, 1.62)
LDL cholesterol (mmol/L)	3.35 (2.66, 3.84)
Triglycerides (mmol/L)	0.98 (0.70, 1.51)
SBP (mmHg)	118 (112, 125)
DBP (mmHg)	74.5 (69.5, 78.0)
sAdiponectin (ng/mL)	3333 (2556, 5626)
sVisfatin (ng/mL)	2.69 (2.31, 3.55)
sTNFα (pg/mL)	0.56 (0.43, 0.72)
AT-Adiponectin (RQ)	0.65 (0.39, 0.97)
AT-Visfatin (RQ)	0.84 (0.58, 1.19)
AT-TNFα (RQ)	1.22 (0.68, 1.66)
sSAT, cm^2^	94.5 (67.3, 124.8)
dSAT, cm^2^	84.5 (50.5, 128.8)
VAT, cm^2^	92.6 (61.2, 149.3)

Values are given as number (proportions), (median 25, 75 percentile) GDR; glucose disposal rate, BMI, body mass index; HDL, high-density lipoprotein; LDL, low-density lipoprotein; SBP, systolic blood pressure; DBP, diastolic blood pressure; s, serum; AT, adipose tissue; RQ, relative quantification; SAT, subcutaneous adipose tissue; dSAT, deep subcutaneous adipose tissue; VAT, visceral adipose tissue.

### Associations Between Circulating and mRNA Expression of Adipokines and the Amount of Abdominal AT

Significant inverse correlations were seen between circulating adiponectin levels and the amount of AT in sSAT, dSAT and VAT (r = -0.266 to -0.358, p<0.05 for all), whereas AT expressed adiponectin was not correlated to either AT compartment. AT expressed TNFα was positively correlated to the amount of AT in sSAT, dSAT and VAT (r=0.323 – 0.386, p<0.05 for all). There was a weak inverse correlation between the amount of VAT and circulating TNFα (r=-0.276, p<0.05), and between the amount of dSAT and AT expression of visfatin (r=-0.265, p<0.05). Circulating visfatin was not correlated to any of the abdominal AT compartments (**Table 2**).

**Table 2:** Correlations (Spearmans rho) between the amount of AT in the different.

	AT expression			Circulating levels		
	Adiponectin	Visfatin	TNFα	Adiponectin	Visfatin	TNFα
sSAT	-0.118	-0.198	0.368**	-0.266*	0.095	0.206
dSAT	-0.220	-0.265*	0.337**	-0.358**	0.092	0.189
VAT	-0.128	-0.137	0.323*	-0.276**	0.181	-0.252*

Abdominal compartments and the measured markers (n=94)

AT, adipose tissue; sSAT, superficial subcutaneous adipose tissue; dSAT, deep subcutaneous adipose tissue; VAT, visceral adipose tissue.

**p < 0.01.

*p < 0.05.

When looking at the aforementioned correlations after dividing the study population into lean and over-weight defined by BMI below or above 25 kg/m^2^, respectively, we found that visfatin RNA expression correlated with sSAT (r=0.407, p=0.035) and TNFα RNA expression correlated inversely with VAT (r=-0.440, p=0.041) in the lean population. In the overweight population, sSAT and dSAT correlated inversely with circulating visfatin (r=-0.314 and -0.325, p<0.03, both) and dSAT correlated inversely with circulating adiponectin (r=-0.286, p=0.038) (**Supplementary Tables 2A, B**).

**Figure 1:**
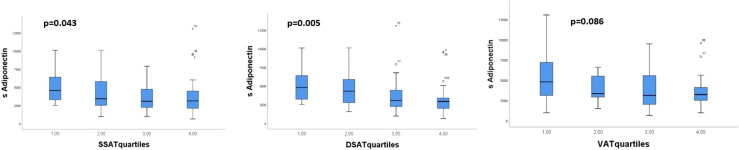
Distribution of circulating adiponectin through quartiles of the abdominal AT compartments sSAT, dSAT and VAT. p-values refer to Kruskal-Wallis test.

**Figure 2:**
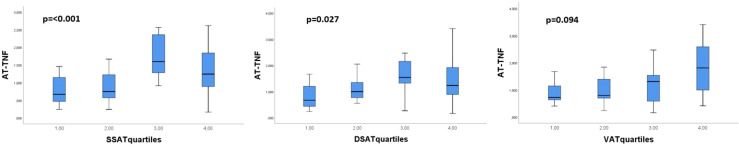
Distribution of genetically expressed TNFα through the abdominal AT compartments sSAT, dSAT and VAT/ Kruskal-Wallis test.

When we divided the amount of AT in sSAT, dSAT and VAT into quartiles, we found that the lowest quartile of sSAT and dSAT had the highest level of circulating adiponectin with a tendency of a gradual decrease in circulating adiponectin levels with increasing amount of AT (p>0.05 for both) (**Figure 1**). The opposite was observed for AT expressed TNFα, with significantly higher levels in the upper compared to the lower quartiles of sSAT and dSAT (**Figure 2**). A similar but statistically non-significant tendency was observed for the distribution of circulating adiponectin and TNFα AT expression, respectively, through VAT quartiles (**Figures 1** and **2**).

### Associations Between AT Expressed and Circulating Adipokines and Glucometabolic Variables 

**Figure 3:**
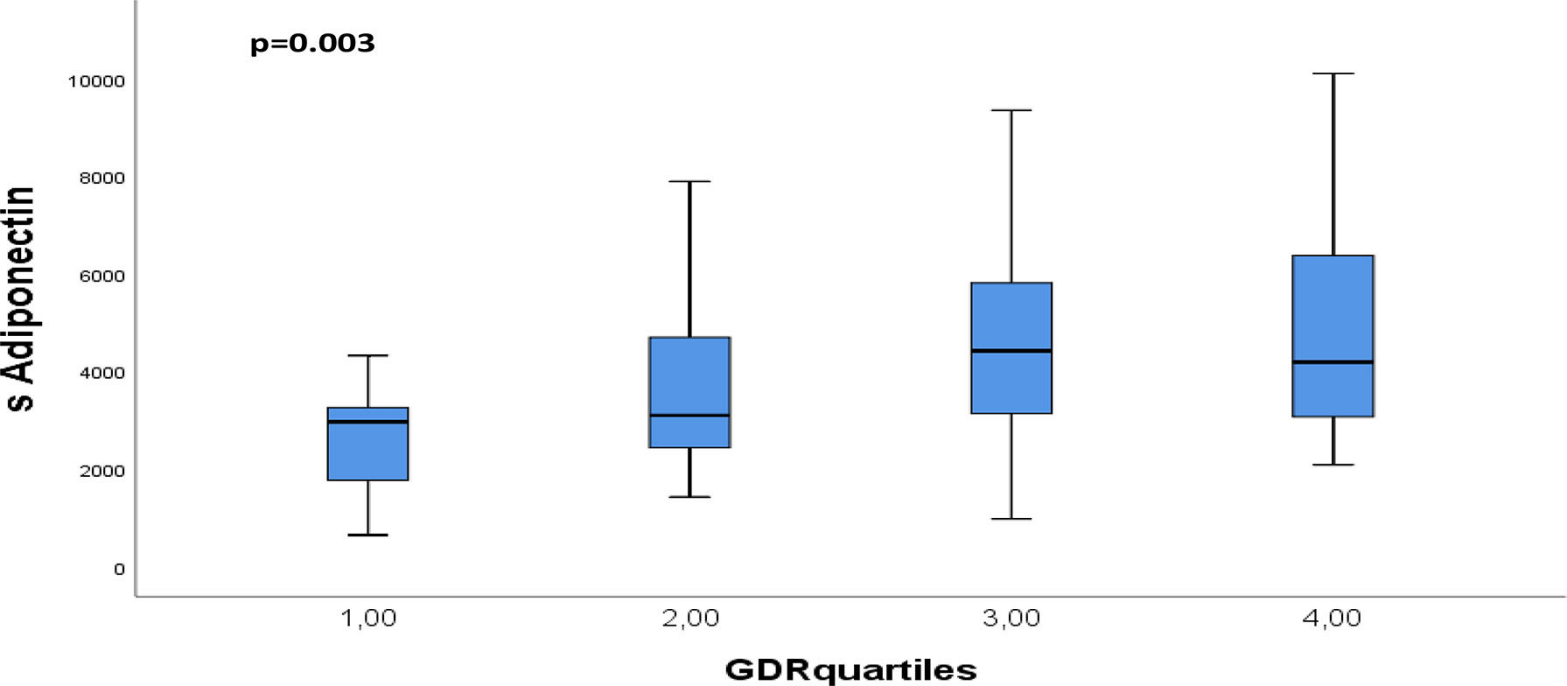
Distribution of circulating adiponectin through quartiles of GDR. p-values refers to Kruskall Wallis test.

After Bonferroni correction, AT expression of visfatin correlated inversely to insulin and C-peptide (r=-0.370 and r=-0.404, p<0.001). Circulating adiponectin correlated inversely to insulin, C-peptide and waist circumference (r=-0.456 to r=-0.373, p<0.001) and positively to GDR (r=0.356, p<0.001) (**Table 3**). When dividing GDR into quartiles, we could observe a gradual increase in serum adiponectin levels in the upper quartiles of GDR (p=0.003) (**Figure 3**).

**Table 3:** Correlations (Spearmans Rho) between glucometabolic variables and the investigated markers.

	AT expression			Circulating levels		
	Adiponectin	Visfatin	TNFα	Adiponectin	Visfatin	TNFα
GDR	r=0.221 p=0.052	r=0.273 p=0.018	r=-0.294 p=0.020	r=0.356 p<0.001*	r=-0.089 p=0.005	r=-0.283 p=0.004
Fasting glucose	r=-0.226 p=0.017	r=-0.201 p=0.079	r=0.164 p=0.195	r=-0.133 p=0.184	r=0.250 p=0.011	r=0.209 p=0.035
HbA1c	r=-0.165 p=0.143	r=-0.048 p=0.678	r=0.116 p=0.360	r=0.020 p=0.843	r=0.022 p=0.824	r=0.013 p=0.900
Insulin	r=-0.309 p=0.006	r=-0.370 p=0.001*	r=0.300 p=0.016	r=-0.456 p<0.001*	r=0.093 p=0.359	r=0.264 p=0.008
C-peptide	r=-0.318 p=0.005	r=-0.404 p<0.001*	r=0.302 p=0.015	r=-0.434 p<0.001*	r=0.134 p=0.184	r=0.263 p=0.008
BMI	r=-0.230 p=0.004	r=-0.282 p=0.014	r=0.344 p=0.005	r=-0.289 p=0.004	r=0.171 p=0.088	r=0.195 p=0.052
Waist	r=-0.197 p=0.084	r=-0.262 p=0.023	r=0.344 p=0.006	r=-0.373 p<0.001*	r=0.173 p=0.087	r=0.275 p=0.006

AT, Adipose tissue.

GDR, Glucose disposal rate; BMI, Body mass index.

*Significant after Bonferroni correction (p = 0.001 by 42 performed associations).

### Correlations Between mRNA Expression of Adipokines and Their Corresponding Circulating Levels

No significant correlations were observed between the AT expressed adipokines and their corresponding circulating levels, except for a statistically significant correlation between AT expressed TNFα and circulating visfatin (r=0.284, p=0.024) (**Supplementary Table 3**).

## Discussion

One of the main findings in our study on healthy middle-aged men was an inverse association between circulating adiponectin levels and the amount of abdominal AT, particularly in dSAT and VAT. There seems to be a unanimous agreement on the beneficial effects of adiponectin on metabolic conditions such as insulin resistance, diabetes and MetS, due to its insulin-sensitizing effect (27). In accordance with this, we could demonstrate circulating adiponectin to be inversely correlated to insulin and C-peptide and positively correlated to insulin sensitivity, assessed by GDR. Previous studies have shown a similar pattern, and considering adiponectin’s beneficial mechanism of action, the findings fits well with the literature (28), and it seems that these mechanisms are similar in healthy subjects.

The deep layers of the AT, in particular the visceral component, has consistently showed an association to dyslipidemia and MetS (29). However, the quantity of VAT and SAT varies between individuals and it seems like their biological effect also are related to their anatomical sites (30). Furthermore, although adiponectin is almost exclusively, produced by AT, the contribution of VAT vs. SAT and dSAT to the circulating adiponectin levels has not been fully understood and data here seems to be somewhat conflicting. From our study, it seems that dSAT is even as important as SAT and VAT. However, *in-vitro* gene expression of adiponectin was shown to be lower in VAT than in SAT, suggesting SAT to be a more important source for adiponectin (31). In a study performed on 783 non-obese, healthy men aged 20-29 years, circulating adiponectin was shown to correlate significantly and inversely to BMI, waist-circumference and to the amount of both VAT and SAT in a univariate analysis, but in a multiple regression analysis only the inverse correlations to SAT remained significant (31). The VAT and its resident macrophages are believed to be more active in the production of pro-inflammatory cytokines, such as TNFα, IL-18 and MCP-1, and less active with adiponectin production (30). These pro-inflammatory cytokines are discussed to be involved in the pathogenesis of metabolic disorders and endothelial dysfunction, partly through inhibition of adiponectin (32). Based on this, one may speculate whether the benefits of reduction in VAT are primarily due to reduced pro-inflammatory cytokine production, whereas the benefits of SAT or dSAT reduction are derived from increased production of beneficial adipokines such as adiponectin. On the other hand, a study on 916 overweight but not affected by obesity (BMI 28.8kg/m2) participants conducted by Jain et al. showed a stronger inverse correlation between adiponectin and the amount of VAT compared to other AT compartments (33).

Low levels of adiponectin in combination with high VAT mass has been associated with insulin resistance, decreased blood glucose control and metabolic disorders (34). When we divided our study population into lean and over-weight, based on BMI below or above 25, respectively, we found that limited correlations between the AT compartments to circulating adiponectin and TNFα RNA expression in either groups, unlike the findings in the total population. The limited correlations found in the subgroups was unexpected, but could possibly be explained by the smaller number of samples in each group, hence under-powering the study. In another study, it was shown that reduction in both SAT and VAT after one year of lifestyle changes with body weight reduction correlated to changes in circulating adiponectin levels and more so for VAT than for SAT (35). Lastly, studies have reported that visceral fat accumulation contributes to low levels of adiponectin (36). The underlying mechanism could be reduced production directly from VAT or, more likely, increased production of inhibiting factors such as TNFα (36).

Another main finding in our study was that TNFα gene expression correlated to the AT amount in all compartments, and particularly in SAT. The known associations between TNFα and reduced insulin sensitivity and MetS are probably mediated through multiple mechanisms, including suppression of insulin receptor and GLUT4, responsible for glucose uptake, and through inhibition of adiponectin (17). Contrary to our results, there seems to be more consistent data on a stronger association between visceral TNFα expression and VAT mass compared to TNFα and other AT compartments, however, unlike our population these study populations were either affected by obesity or had other underlying metabolic diseases (37, 38). This poses the question of whether there is a shift from SAT to VAT as the main source for TNFα expression as the metabolic disease transpires. However, even though TNFα expression has been shown to associate with VAT mass, net secretion of TNFα from VAT into the circulation has not yet been documented.

Gene expression and circulating levels of visfatin was limited associated with the amount of AT in sSAT, dSAT and VAT in our population. It has been suggested that visfatin is primarily produced by VAT and associates with intra-abdominal fat mass, but not with SAT (15). Visfatin seems to play an important role in obesity induced insulin-resistance (15). We did however, observe an inverse relationship between visfatin gene expression to insulin and C-peptide. It has been proposed that visfatin production might potentially be low in the lean state and that intra-abdominal obesity leads to increased visfatin production, which simultaneously increases with obesity, but also changes the properties of visfatin, giving it more insulin-mimetic qualities (15). This could to some extent, explain our findings with inverse correlations to insulin and C-peptide.

### Limitations

Our study was a cross sectional study, thus, we cannot, with any degree of certainty, say anything about causality and the interpretation of the results therefore remains speculative. We have primarily investigated associations between the adipokines and the amount of AT in the different abdominal compartments, in order to gain a better understanding of the source and their role in developing/protecting against metabolic disease. It should also be emphasized that gluteal SAT samples were used for the gene expression analyses. As differences between gluteal and abdominal SAT have been demonstrated, our results cannot be directly transferred to SAT in general (39, 40). Another major limitation is the lack of females in our study; hence, the applicability to the general population is limited. The study design confines us from making any power calculations.

The strengths of our study are the CT measurement of AT distinguishing the different compartments and that insulin sensitivity was assessed by glucose clamp, which is the gold standard.

## Conclusion

In this population of healthy, slightly overweight men, circulating levels of adiponectin, associated inversely with the amount of AT in all abdominal AT-compartments and to some glucometabolic variables. This may indicate that high amount of AT with simultaneous low levels of circulating adiponectin, could potentially be early signs of increased risk of developing metabolic disorders. The observed association between TNFα gene expression and the amount of abdominal AT, particularly in sSAT and dSAT, could indicate that SAT from the gluteal region also contributes to an increased pro-inflammatory state.

## Data Availability Statement

The original contributions presented in the study are included in the article/**Supplementary Material**. Further inquiries can be directed to the corresponding author.

## Ethics Statement

The studies involving human participants were reviewed and approved by Regional Committees for Medical and Health Research Ethics. The patients/participants provided their written informed consent to participate in this study.

## Author Contributions

HZ conducted the study and was responsible for the statistical analyses and drafting the manuscript. TA was responsible for the main randomized trial involving recruitment, contributed to the study protocol, acquired data and contributed to the intellectual content of the manuscript. SÅ was responsible for laboratory analyses and contributed in the discussion of the manuscript. HE contributed to the manuscript. RB contributed to the manuscript. IS contributed to the intellectual content, interpretation of the results and drafting the manuscript. TB was involved in drafting of the manuscript, interpretation of the results and contributed to the intellectual content. All authors read and approved the final version.

## Funding

The work was supported by Stein Erik Hagens Foundation for Clinical Heart Research, Oslo, Norway.

## Conflict of Interest

The authors declare that the research was conducted in the absence of any commercial or financial relationships that could be construed as a potential conflict of interest.

## Publisher’s Note

All claims expressed in this article are solely those of the authors and do not necessarily represent those of their affiliated organizations, or those of the publisher, the editors and the reviewers. Any product that may be evaluated in this article, or claim that may be made by its manufacturer, is not guaranteed or endorsed by the publisher.

## Supplementary Material

Supplementary Table 1 Methods. AT, adipose tissue; SAT, Subcutaneous adipose tissue; sSAT, superficial subcutaneous adipose tissue; dSAT, deep subcutaneous adipose tissue; VAT, visceral adipose tissue; mRNA, messenger RNA; RT-PCR, Real-time polymerase chain reaction.

Supplementary Table 2
**(A)** Abdominal compartments and the measured markers in the lean population (BMI < 25) (n = 40). AT, adipose tissue; sSAT, superficial subcutaneous adipose tissue; dSAT, deep subcutaneous adipose tissue, VAT, visceral adipose tissue. *p < 0.05**. (B)** Abdominal compartments and the measured markers in the overweight population (BMI >25) (n = 62). AT, adipose tissue; sSAT, superficial subcutaneous adipose tissue; dSAT, deep subcutaneous adipose tissue; VAT, visceral adipose tissue. *p < 0.05

Supplementary Table 3 Correlation (Spearmans Rho) between genetically expressed adipokines and corresponding circulating levels. AT; adipose tissue. s; serum levels. *p < 0.05.
