# Supplementary material for: Abdominal Adipose Tissue Associates With Adiponectin and TNFα in Middle-Aged Healthy Men
Source: Front Endocrinol (Lausanne). 2022 Jul 7;13:874977. doi: 10.3389/fendo.2022.874977 (PMC9301307; doi:10.3389/fendo.2022.874977)
Supplement: Supplementary Table 1 — Methods. AT, adipose tissue; SAT, Subcutaneous adipose tissue; sSAT, superficial subcutaneous adipose tissue; dSAT, deep subcutaneous adipose tissue; VAT, visceral adipose tissue; mRNA, messenger RNA; RT-PCR, Real-time polymerase chain reaction. [file Table_1.docx]

**Supplementary Table 1**

Abdominal compartments and the measured markers in the lean population (BMI <25) (n=40)

|  | AT expression | | | | |  | Circulating levels | | |
| --- | --- | --- | --- | --- | --- | --- | --- | --- | --- |
|  | Adiponectin | | Visfatin | TNFα | |  | Adiponectin | Visfatin | TNFα |
| sSAT | r=0.315  p=0.109 | r=0.407*  p=0.035 | | | r=0.178  p=0.428 |  | r=-0.234  p=0.164 | r=-0.047  p=0.784 | r=0.042  p=0.803 |
| dSAT | r=0.073  p=0.717 | r=0.263  p=0.186 | | | r=0.163  p=0.469 |  | r=0.187  p=0.269 | r=-0.018  p=0.916 | r=-0.065  p=0.704 |
| VAT | r=0.321  p=0.102 | r=0.198  p=0.323 | | | r=-0.440*  p=0.041 |  | r=-0.243  p=0.148 | r=-0.157  p=0.354 | r=0.032  p=0.852 |

AT; adipose tissue, sSAT; superficial subcutaneous adipose tissue, dSAT; deep subcutaneous adipose tissue, VAT; visceral adipose tissue

**p<0.01

*p<0.05

Abdominal compartments and the measured markers in the overweight population (BMI >25) (n=62)

|  | AT expression | | | | |  | Circulating levels | | |
| --- | --- | --- | --- | --- | --- | --- | --- | --- | --- |
|  | Adiponectin | | Visfatin | TNFα | |  | Adiponectin | Visfatin | TNFα |
| sSAT | r=-0.229  p=0.145 | r=-0.192  p=0.234 | | | r=.0.115  p=0.504 |  | r=-0.138  p=0.325 | r=-0.314*  p=0.022* | r=0.029  p=0.835 |
| dSAT | r=-0.302  p=0.052 | r=-0.306  p=0.055 | | | r=-0.157  p=0.360 |  | r=-0.286*  p=0.038 | r=-0.325*  p=0.018 | r=-0.032  p=0.819 |
| VAT | r=-0.139  p=0.379 | r=0.103  p=0.526 | | | r=0.150  p=0.382 |  | r=-0.111  p=0.428 | r=0.022  p=0.875 | r=0.110  p=0.435 |

AT; adipose tissue, sSAT; superficial subcutaneous adipose tissue, dSAT; deep subcutaneous adipose tissue, VAT; visceral adipose tissue

**p<0.01

*p<0.05

Supplementary table 2 – Method

| **Measures** | **Material** | **Region** | **Region** | **Region** | **Method** |
| --- | --- | --- | --- | --- | --- |
| mRNA expression of adiponectin, visfatin and TNFα | Gluteal SAT |  |  |  | RT-PCR |
| Amount of abdominal AT cm^2^ |  | sSAT | dSAT | VAT | CT scan |
| Circulating levels of  adiponectin, visfatin and TNFα | serum |  |  |  | ELISA methods |

AT; adipose tissue, SAT; Subcutaneous adipose tissue, sSAT; superficial subcutaneous adipose tissue, dSAT; deep subcutaneous adipose tissue, VAT; visceral adipose tissue, mRNA; messanger RNA, RT-PCR; Real-time polymerase chain reaction

Supplmentary table 3 – Correlations between mRNA expression of Adipokines and their corresponding circulating levels

|  | AT adiponectin | AT Visfatin | AT TNFα |
| --- | --- | --- | --- |
| s Adiponectin | r=0.188  p=0.097 | r=0.218  p=0.059 | r=-0.121  p=0.345 |
| s Visfatin | r=-0.143  p=0.207 | r=-0.089  p=0.445 | r=0.284  p=0.024* |
| s TNF | r=-0.178  p=0.117 | r=0.097  p=0.405 | r=0.116  p=0.367 |

AT; adipose tissue

s; serum levels

* p<0.05
